# Saliva proteomics updates in biomedicine

**DOI:** 10.1186/s40709-019-0109-7

**Published:** 2019-12-12

**Authors:** Katerina R. Katsani, Dimitra Sakellari

**Affiliations:** 1 Department of Molecular Biology & Genetics, Democritus University of Thrace, Alexandroupolis, Greece; 2 Department of Preventive Dentistry, Periodontology and Implant Biology, School of Dentistry, Aristotle University of Thessaloniki, Thessaloniki, Greece

**Keywords:** Saliva, Proteomics, Disease

## Abstract

In the years of personalized (or precision) medicine the ‘omics’ methodologies in biomedical sciences—genomics, transcriptomics, proteomics and metabolomics—are helping researchers to detect quantifiable biological characteristics, or biomarkers, that will best define the human physiology and pathologies. Proteomics use high throughput and high efficiency approaches with the support of bioinformatic tools in order to identify and quantify the total protein content of cells, tissues or biological fluids. Saliva receives a lot of attention as a rich biological specimen that offers a number of practical and physiological advantages over blood and other biological fluids in monitoring human health. The aim of this review is to present the latest advances in saliva proteomics for biomedicine.

## Background

This mini review aims at providing an outline of the prospective clinical applications of salivary proteomics in a large spectrum of human diseases with a special focus to the bibliography over the last 5 years. For this purpose, we performed PubMed literature searches in NCBI using various combinations of keywords, such as Saliva AND Omics and limited our search to the references in the years 2014–2019 and with a few exceptions to large scale studies only.

**Fig. 1:**
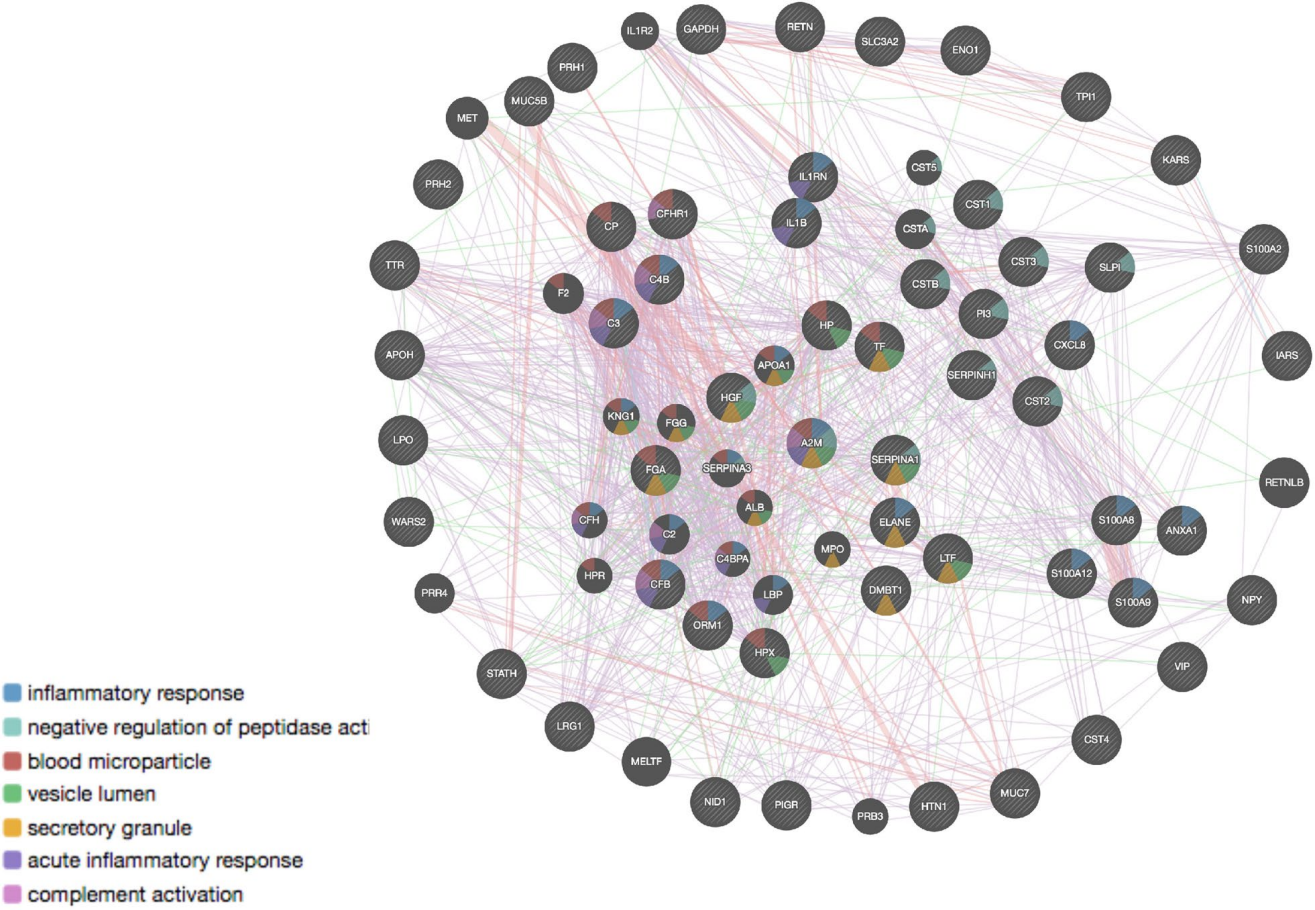
Pathway analysis using GENEMANIA [90]. The seven most relevant pathways are shown (nodes are colored accordingly). The network was generated taking into account the co-expression, physical interactions, pathway and genetic interaction networks (edges). Protein entries used for the network are given as Additional file 1

## Introduction

Saliva is a physiological fluid serving primarily as an antibacterial, antifungal and lubricant agent for digestion of food. Its antiseptic and protective properties were known in Ancient Greek Medicine but more recently its value as a non-invasive diagnostic tool in personalized medicine is also being recognised.

Although saliva is considered a less likeable fluid compared with blood and tears [(“spittle”), Proto-Indo-European salw-, sal- (“dirt, dirty”)], the process of obtaining saliva is the easiest and least invasive (e.g. in comparison to blood) [1–3]. Saliva sampling is not invasive and entails less discomfort for the patient, therefore it offers a more well-accepted alternative to blood testing for less cooperative populations such as institutionalized individuals and infants [4, 5]. Moreover, saliva collection presents minimal risk of infection for the personnel and its sampling and storage require very basic equipment. Therefore, it can be easily performed in lab settings of developing countries and by untrained personnel [6]. Saliva can be collected as individual secretions from the major and minor salivary glands, as well as whole saliva in unstimulated (resting) or stimulated conditions, with several means of stimulating secretion, usually via the chewing of various substances [7–9]. A number of standardized highly effective collection devices are now available [8]. Importantly, the saliva collection method can influence the salivary flow, as well as composition and integrity of the samples and has to be carefully selected [9–11].

The flow rate and composition of saliva are effectively regulated by the autonomous nervous system, and are dependent on signalling by neuropeptides and intracellular calcium [12]. They are further influenced by a number of factors such as age, circadian rhythm, psychological factors such as pain and stress, and any factors such as some medication and diseases (oral and systemic) affecting the physiology of the salivary glands [6]. The salivary proteome varies from birth to adolescence and it is necessary to take age into account in data referencing [13]. Advances in collection and analytical methods enabled more reliable results even from neonates making saliva a promising assessment tool in paediatrics [5, 14].

Apart from small molecules such as urea, and electrolytes, saliva largely contains the human oral microbiome and food debris. The human oral microbiome is one of the most diverse of the human body and constitutes a dynamic entity that contributes more than 2000 microbial proteins from more than fifty bacterial genera to the saliva proteome [15]. Certain collection methods may contribute to larger numbers of bacteria in the sample, nevertheless this can be minimized. Bacteria from teeth and gingival crevices normally make only a small contribution while the various oral hygiene practices have similar effects on bacterial output into saliva [11].

Less than 1% of saliva that is not water, is rich in lubricant proteins, such as mucins, proline-rich glycoproteins and elements of the innate immunity system that offer antimicrobial activity from microbial proteases [16, 17]. Although a high variability in protein content has been observed depending on collection time, sex, age, and pathological conditions, the typical protein concentration of saliva is 0.7–2.4 mg·mL^−1^. Compared to plasma saliva is a more dilute and less complex biofluid with four-fold less total protein content. Protein concentrations can be 1000-fold less (than in plasma) posing technological challenges in diagnostics. Saliva can contain proteins infiltrated from blood usually from inflamed gingivae and microinjuries [9]. Interestingly, 30% of the saliva proteome is shared with the blood plasma proteome [18, 19]. Many of these proteins are post-translationally modified with glycosylation, acetylation, phosphorylation, and proteolysis, while a large number of peptides is present due to higher degradation processes in the oral cavity [20, 21]. Special care is recommended in sample collection processing and storage in order to minimize degradation processes altering proteome composition [9]. Moreover, saliva contains a number of hormones and growth factors. Interestingly, the levels of estrogen and progesterone in the saliva of premenopausal women fluctuate according to the menstrual cycle phases and correlate with their fluctuation in blood serum [22].

The diagnostic potential of saliva was demonstrated already in 1990s [23]. A decade later the American National Institute of Dental and Craniofacial Research (NIDCR) funded a collaborative effort to support scientific research on clinical applications for saliva and in this direction the University of California at Los Angeles (UCLA) developed a data repository, and a web resource, “The Salivaomics Knowledge Base” (SKB; http://www.skb.ucla.edu/) aiming to centralize human salivary omics data [3].

This mini review aims to provide an outline of the prospective clinical applications of salivary proteomics in a large spectrum of human diseases (Table 1). For this purpose, we performed PubMed literature searches in NCBI using various combinations of keywords, such as Saliva AND Omics and limited our search to the references of the years 2014–2019 and with a few exceptions to large scale studies only.

**Table 1:** An outline of the prospective clinical applications of salivary proteomics in a large spectrum of human diseases

Disease	Reference nos.	Proteins in the text	Results
Oral diseases			
Periodontitis	[39]	MMP-8	Correlation with the severity of periodontitis
Periodontitis	[40]	Apolipoprotein H	Discriminatory factor for chronic and aggressive periodontitis
Periodontitis	[41]	Trappin-2 and cytokine IL-1β	Anti-protease/proinflammatory cytokine imbalance
Periodontitis	[42]	S100A8 and S100A9	Candidate biomarkers for periodontitis
Periodontitis	[43]	ANXA1	Potential early biomarker for gingival inflammation during pregnancy
Periodontitis	[44]	HGF	Positive correlation with periodontitis progression and smoking habits, and monitoring response to therapy
Periodontitis	[45]	VIP and NPY	Potential gender-specific salivary biomarkers for periodontitis
Oral cancer			
OSCC	[49]	Complement proteins, CFB, C3, C4B	Predictive biomarkers related to risk of development OSCC
OSCC	[49]	SERPINA1 and LRG1	Predictive biomarkers related to risk of development OSCC
OSCC	[50]	SERPINA1, CFH, FGA	Potential salivary biomarkers for OSCC diagnosis
OSCC	[51]	IARS, KARS, WARS, YARS	Elevated levels in tumour interstitial fluids
OSCC	[51]	NID1	Potential OSCC biomarker
OSCC	[53]	SLPI	Decreased in premalignant lesion and OSCC lesion tissues
OSCC	[54]	SLC3A2, S100A2, IL1RN	Potential OSCC biomarkers
OSCC	[55, 56, 58],	IL8, IL1beta, Resistin	Potential OSCC biomarkers
Other cancer types			
Gastric cancer	[59]	CSTB, TPI1, and DMBT1	Discriminatory biomarkers in cancer cases
Infiltrating ductal carcinoma	[60]	α2-macroglobulin and ceruloplasmin	Upregulated
Autoimmune diseases			
cGVHD	[61]	Lactotransferrin lactoperoxidase	Reduced levels
cGVHD	[61]	IL-1ra, cystatin B	Potential diagnostic biomarkers
Sjogren’s syndrome (SS)	[62]	MUC5B and MUC7	Altered glycosylation and sulfation patterns
Sjogren’s syndrome (SS)	[64]	Calcium-binding proteins, defence-response proteins, proteins involved in apoptotic regulation, stress- response proteins and cell motion- related proteins	Increased in SS patients
Sjogren’s syndrome (SS)	[68]	S100A8/A9	Potential biomarkers for SS patients with lymphoma or at higher risk of lymphoma
Sjogren’s syndrome (SS)	[69]	S100 proteins	Potential early biomarkers for primary SS
Other systemic diseases			
Systemic diseases and periodontitis	[72]	Visfatin	Putative biomarker for both
Periodontitis and type 2 diabetes	[73]	Ferritin, hepcidin	Positive correlation between salivary and serum ferritin and low salivary hepcidin levels
Multiple sclerosis	[71]	S-type cystatins	Altered glycosylation and oxidation levels
Infectious diseases			
Zika virus	[75]	Viral proteins	Saliva may be a repository for free Zika virus particles and infected cells
Dengue virus	[76]	Anti-NS1 antibodies	Detected with comparable sensitivity in plasma and saliva
HBV and HCV	[77]	C3, alpha(1)-acid and alpha(2)-acid glycoproteins, haptoglobin, serotransferrin, ceruloplasmin	Potential biomarkers
HCC	[77]	Hemopexin, transthyretin, GADPH, alpha- enolase, and cystatin C	Their monitoring in saliva could substitute blood tests
Rare diseases			
SAPHO	[80]	S100A12	Potential biomarker
Wilson disease	[81]	S100 A9 and S100 A8	Oxidation levels could monitor disease progression
Neurological diseases			
Autism spectrum disorders	[2]	Statherin, histatin 1, and acidic proline-rich proteins	Decreased levels
Autism spectrum disorders	[2]	Prolactin-inducible protein, lactotransferrin, Ig kappa chain C region, Ig gamma- 1 chain C region, Ig lambda-2 chain C regions, neutrophil elastase, polymeric immunoglobulin receptor and DMBT1	Elevated levels

The seven more relevant pathways affected are depicted in a GENEMANIA network in Fig. 1 [90]

### Technology and methodological approaches

The key technology of proteomics is mass spectrometry (MS) supported by bioinformatics tools for data acquisition and management [24]. However, the most critical steps in every proteomic experiment are sample isolation and preparation preceding the analysis [9]. A number of classical biochemical techniques in proteomics, including gel electrophoresis, liquid chromatography, and microarrays, are used for sample stabilization, fractionation, and enrichment for groups of proteins or modifications before analysis by mass spectrometry [25, 26]. Saliva collection and processing is very critical as it can significantly influence its composition [9]. Moreover, controls and samples between studies need to be matched in terms of age of individuals, time and method of sampling to allow for reliable results and the correct comparison among studies [27]. Like serum, saliva, is a complex mixture containing proteins with a large range of concentrations, more than ten orders of magnitude. In these samples, protein biomarkers are present in very low amounts and are easily degraded, while the presence of exogenous proteins, e.g. the microbiome can further influence the reproducibility of the results [28]. The use of immunodepletion protocols for the removal of the most abundant albumins and immunoglobulins [29, 30] and the development of re-usable and/or low cost devices for sample preparation that give reliable and reproducible results [31] has helped the in-depth analysis of biomarkers [30].

Lately, technological advances have upgraded the mass-spectrometry platforms from the DDA (Data-Dependent Acquisition) mode to the DIA (Data-Independent Acquisition) mode and from the Discovery (or Identification) to Targeted (and Quantitative) acquisition mode of functioning [32]. The DDA mode stochastically detects only the most abundant peptides and misses the rest, while the DIA mode repeatedly selects mixtures of peptide species within large, predefined mass ranges of fragmented ion scans. DIA is more likely to sample all peptides within the selected mass ranges and it is not predisposed to detect only the most abundant, masking the discovery of peptide traces and often significant biomarkers [33].

The targeted acquisition mass spectrometry strategies, SRM (selected reaction monitoring), MRM (multiple reaction monitoring) and PRM (parallel reaction monitoring), can be effectively used in the precise and reproducible quantification of hundreds of proteins of low abundance poised for clinical use [32]. While all of the above three strategies give promising results in the field of biomarker discovery, SRM is the most promising to be introduced in routine clinical settings for diagnostic and prognostic purposes. SRM has been dubbed as the “mass spectrometrist’s ELISA” as the most likely technique to replace ELISA in the future. SRM targets proteins using a predetermined assay with high sensitivity and selectivity, without need for immunoassays. Compared to ELISA it offers a higher degree of multiplexing (up to 100 proteins can be concomitantly quantified) and it enables the quantification of species that cannot be easily distinguished using antibodies (protein isoforms, or post-translationally modified proteins), having as only prerequisite a mass shift [34]. More recently, the field of saliva proteomics and diagnostics has been additionally profited from the development of novel technologies for electrochemical detection apparatuses and point-of-care diagnostics. The “Collaborative Oral Fluid Diagnostic Research Centre” at UCLA, has developed an integrated POC (point-of-care) multiplexing saliva-based platform for oral cancer detection. With this setup, one can detect both salivary proteins and nucleic acids and measure up to eight different biomarkers in a single test in less than 15 min under ambient conditions [35]. Finally, proteomic analysis of salivary exosomes—that are cell-derived vesicles, 30–100 nm in diameter, with an important role in intracellular communication—could play a promising role in the identification of diagnostic and therapeutic biomarkers for systemic and oral diseases [36, 37].

### Oral diseases-periodontitis

Periodontitis is a complex multi-factorial, immune-inflammatory disease that can be asymptomatic and unnoticed for years. In periodontitis, the teeth supportive tissue and bone is compromised due to the elicit of host inflammatory and immune mechanisms resulting in the deepening of the periodontal pocket, and finally in tooth loss [38]. Periodontitis is expensive to treat, therefore newly discovered biomarkers could be used for onset risk determination, treatment planning and prognosis of disease progression. Traditional clinical assessment criteria are error-prone and inadequate for disease prognosis and risk-assessment. A considerable number of studies have focused on the correlation of disease severity (mild-moderate-severe) with the presence of inflammation molecules, cytokines, prostaglandins, and the levels of proteases in saliva and serum. To this end, statistically significant elevated levels of matrix metalloproteinase-8 or collagenase MMP-8 in saliva has been correlated with the severity of the disease using an immunofluorometric assay [39]. Apolipoprotein H, a glycoprotein involved in a number of physiological processes, was suggested as a discriminatory factor for chronic and aggressive periodontitis, among 35 candidate proteins in an MRM study [40]. Trappin-2, an anti-inflammatory serine protease inhibitor identified in several chronic infections, was decreased in periodontitis while the cytokine IL-1β levels were increased compared to healthy individuals levels, demonstrating an anti-protease/proinflammatory cytokine imbalance [41]. A shotgun proteomics analysis indicated that higher levels of salivary calcium‐binding proteins, S100A8 and S100A9, could be candidate biomarkers for periodontitis [42]. Bacterial colonization of the periodontal pocket, genetic predisposition, lifestyle (smoking) as well as physiological factors (such as pregnancy, stress or diabetes mellitus) contribute to periodontitis development [38]. Indeed, annexin-1 (ANXA1), an anti-inflammatory protein, was identified in gingival tissue as a potential early screening salivary biomarker for gingival inflammation during pregnancy [43]. The levels of hepatocyte Growth Factor (HGF), a multifunctional cytokine, were found to positively correlate with periodontitis progression and smoking habits. A significant reduction in HGF levels was observed after non-surgical periodontal therapy suggesting that HGF could be used to monitor response to periodontal therapy [44]. Vasoactive Intestinal Peptide (VIP), and Neuropeptide Y (NPY) in saliva (but interestingly not in serum) could potentially be gender-specific salivary biomarkers for periodontitis, regardless of psychological stress [45]. Concluding, a review [46] gives an overall account of the salivary protein biomarkers identified in oral diseases.

### Oral cancer

Oral squamous cell carcinoma (OSCC) is a concern for populations worldwide. Cultural habits, such as smoking and alcohol consumption are major risk factors while synergism with viral infections is not always evident [47]. Diagnosis is mostly based on endoscopic examination using imaging techniques combined with biopsy for histological analysis, but it is often delayed until later stages, as lesions are usually asymptomatic. This suggests that early screening methods can support preventive treatment [48]. A targeted proteomics SRM study revealed a number of predictive biomarkers related to the risk of development OSCC, namely complement proteins, CFB, C3, C4B, Alpha-1-antitrypsin protein SERPINA1, and LRG1, a protein identified before in the serum and tumours of cancer patients, all found with differentiated expression in OSCC [49]. SERPINA1 along with complement factor H (CFH), fibrinogen alpha chain (FGA), were also selected as potential salivary biomarkers for OSCC diagnosis in a quantitative study with different ethnic populations [50]. Interestingly, the same research group in an independent study (with an independent panel of patients and using a different peptide quantification approach) discovered among others elevated levels of nidogen-1 (NID1) and serpin H1 (SERPINH1) along with proteins of the aminoacyl-tRNA biosynthesis pathway (IARS, KARS, WARS, and YARS) when they examined tumor interstitial fluids (TIF) in comparison with fluids from adjacent noncancerous (NIF) tissues. NID1, a protein that mediates extracellular matrix assembly, was further validated in saliva as a potential OSCC biomarker, using two immunoassays, but not SERPINH1 [51]. Other studies aimed at the discovery of prognostic signatures for improved treatment effectiveness [52]. A promising diagnostic molecule appears to be the secretory leukocyte protease inhibitor (SLPI), a secreted serine protease inhibitor with anti-inflammatory and modulatory functions on immunological responses and cell proliferation. By contrast to other types of cancers, in which higher SLPI expression levels correlated with worse clinical outcome [29], SLPI was found in decreased levels in both oral premalignant lesion tissue and OSCC lesion tissues compared to healthy normal tissue [53]. An independent quantitative proteomics study identified another three potential biomarkers among 246 differentially expressed proteins between healthy individuals and OSCC patients. The three proteins, solute carrier family 3 member 2 (SLC3A2), S100 calcium‐binding protein A2 (S100A2), and interleukin‐1 receptor antagonist protein (IL1RN) could be validated in an independent study with high specificity [54]. Additionally, a number of studies profiled the saliva proteome and secretome of OSCC and healthy subjects in order to function as a reference for biomarker discovery [30, 49, 53–58]. In these studies certain proteins stood out as possible biomarkers, such as IL8, IL1beta and the adipokine marker resistin, but they failed to be consistently validated by all research groups, pointing to variations between populations of different ethnic origins or habits and highlighting the necessity for development of standardized protocols for sample collection and treatment [58].

### Other cancer types

The necessity for non-invasive, reliable biomarkers is very important in cancer cases. Indicatively, gastric cancer incidents, are very diverse in terms of histopathology, therefore, screening and surveillance are necessary. In a 2016 study, three (out of 48) proteins identified in saliva samples and validated by ELISA could potentially differentiate patients from healthy subjects. The combination of these three proteins, namely CSTB (cystatinB), TPI1 (triosephosphate isomerase), and DMBT1 (deleted in malignant brain tumour 1 protein) could distinguish gastric cancer cases with high accuracy [59]. In the case of breast cancer, proteomics assays could minimize the need for invasive procedures to discriminate for malignant or benign pathologies. The saliva and serum proteome have been compared between patients with infiltrating ductal carcinoma and a healthy control group. Differentiated proteins between healthy and patient groups were immune, transport and signalling pathways members [60]. In fact, α2-macroglobulin and ceruloplasmin were two of those proteins found upregulated in both fluids, whose presence linked to breast cancer cases before. Interestingly, a lung cancer study has shown that an approximate 80% of salivary exosomal proteins were shared with serum exosomes and that the saliva proteome of patients was 40% different than that of the healthy group suggesting that saliva could replace blood screening methods. Ninety-seven proteins were unique to lung cancer and one-third of those were linked to known pathways related to cancer metastasis and proliferation, a fact that demonstrated the potential for identifying biomarkers for lung cancer in saliva [37].

### Autoimmune diseases

Oral mucosa is the second tissue after the skin that can deteriorate in patients with chronic graft-versus-host disease (cGVHD), a fatal immunological condition affecting multiple tissues of transplant recipients. The damage of salivary glands causes xerostomia, which together with reduced salivary immunoglobulin production increases the patient’s susceptibility to oral infections. Biomarkers that could distinguish cGVHD from other autoimmune or inflammatory conditions would be very useful as diagnostic tools, as clinical symptoms in these conditions may be misleading. Among the identified proteins reduced levels of two antimicrobial proteins, salivary lactotransferrin and lactoperoxidase, were found in more than one study. More significantly, IL-1ra (interleukin-1 receptor antagonist) that blocks IL-1 signalling and cystatin B, a protease inhibitor and regulatory molecule, had significantly altered expression in association with oral cGVHD [61]. Both proteins have been associated with chronic inflammation and presented with good discriminatory power especially for early diagnosed patients pointing to the fact that they may be used as a promising diagnostic tool.

Sjogren’s syndrome (SS) is an autoimmune exocrinopathy attacking the salivary and lacrimal glands. Its most common oral manifestation is xerostomia but in combination with the development of malignancies, such as non-Hodgkin’s lymphoma, common in autoimmune diseases, it leads to more severe symptoms. In SS patients, the oral mucosa moisturizers, mucins MUC5B and MUC7 appeared in similar levels between patients and healthy subjects but showed reduced glycosylation, and sulfation, two modifications that correlate well with xerostomia in SS patients [62, 63]. Other categories of proteins that were considerably increased in SS patients compared to healthy subjects were calcium-binding proteins, defence-response proteins, proteins involved in apoptotic regulation, stress-response proteins and cell motion-related proteins [64]. Saliva proteomics revealed biomarker signatures that in combination with minor salivary gland biopsy could potentially be used for the diagnosis and classification of primary SS [65], while in another study could differentiate patients with rheumatoid arthritis from SS patients [66]. A panel of autoantibodies that are progressively up-regulated could be potentially used as predictive biomarkers for the progression of primary SS to mucosa-associated lymphoid tissue (MALT) lymphoma [67], while calcium‐binding proteins S100A8/A9 levels in parotid saliva (and not whole saliva) were found to be discriminatory for SS patients with lymphoma or at higher risk of lymphoma [68]. An interesting LC-coupled SWATH-MS study (Liquid Chromatography coupled to a data-independent sequential window acquisition of all theoretical fragment ion spectra study) was used in an effort to search for salivary proteomic biomarkers in primary SS specific subsets. Affected proteins apart from the normal saliva constituents like proline-rich proteins and cystatins that were reduced, related to antimicrobial and in inflammatory response pathways. The family of the S100 proteins were distinguished as potential primary SS early biomarkers for primary SS salivary gland impairment or inflammation, that warrant further validation in larger cohorts [69]. Katsiougiannis and Wong published in 2016 a review that highlights the most significant and promising findings of salivary proteomics in the context of primary SS [70].

### Other systemic diseases

Saliva proteomics can offer easier monitoring of the patient’s physiology (compared to blood testing), as well as prognostic and diagnostic biomarkers that could be used to improve the quality of the patients’ life and reduce the financial burden on state health systems in chronic diseases such as multiple sclerosis and diabetes [7, 71]. The circulating adipokine visfatin levels (in serum or saliva) were found to correlate well with inflammatory disorders such as diabetes mellitus, cardiovascular disease, and periodontitis and linked periodontitis with systemic diseases [72]. In the same line, the highest positive correlation observed between salivary and serum ferritin and low hepcidin levels in periodontitis and type 2 diabetes mellitus could provide a reference for body iron load [73]. Multiple sclerosis is a chronic inflammatory disease of the central nervous system without a single diagnostic test so far. A recent proteomic study of multiple sclerosis patient’s saliva showed, among others, a decreased level of oxidation of S-type cystatins, which may be attributed to the applied therapy, as well as altered glycosylation levels, reported previously for various diseases [71]. Such studies hold promise for improved disease monitoring. For a systemic review on potential proteomic biomarkers of systemic lupus erythematosus, an autoimmune disease that affects multiple organs, and are not described here, readers could refer to [74].

### Infectious diseases

Blood sampling imposes cultural, hygiene and logistical challenges in developing countries which are the most affected by infectious diseases. The use of saliva instead of blood could circumvent these sampling issues if only infectious agents or signalling molecules could be detected in it. Especially, viruses require more frequent sampling because they can remain in a latent condition for years, therefore, the monitoring of viruses in saliva could be more relevant. An example is the recent case of Zika virus, a mosquito-borne flavivirus, associated with complications of pregnancy, malformations in infants and neuropathy and myelitis in adults (http://www.who.int/news-room/fact-sheets/detail/zika-virus). A study in a survivor mother and her two newborn babies has shown that saliva may be a repository for both free Zika virus particles and cells infected with Zika virus that may be latent in the salivary glands [75]. Dengue virus is the cause of another painful and debilitating mosquito-borne disease. Anti-Dengue antibodies were detected with comparable sensitivity in plasma and saliva, therefore testing of anti-NS1 viral protein antibodies could also be used in cases where blood sampling is cumbersome [76]. Hepatitis B and C viruses (HBV and HCV) infections are important causes of cirrhosis and hepatocellular carcinoma (HCC) in patients worldwide. Due to their long latent phase, infected individuals may remain undiagnosed, leading to the continuing spread of these infections, especially among drug users. A reduced number of MS/MS spectra for complement C3, alpha(1)-acid and alpha(2)-acid glycoproteins, haptoglobin, serotransferrin, and ceruloplasmin, was observed in a study that differentiated the (HCV or HBV) infected from the healthy groups. In the same study, the HCC serum biomarker candidates—hemopexin, transthyretin, GADPH, alpha-enolase, and cystatin C—were also detected showing the potential of monitoring saliva instead of blood samples [77]. Finally, a number of studies aimed to identify discriminating patterns among patient groups and investigate their potential role in the discovery of biomarkers of HCC and cirrhosis targeted the glycoproteins in the serum of HCC patients and more recently in the saliva of hepatitis infected and HCC patients [78, 79].

### Rare diseases

Saliva proteomics could contribute to the diagnosis and monitoring of rare diseases, which often lack a specific test. SAPHO is a rare disease in the spectrum of bone autoinflammatory diseases, such as rheumatism, which poses a diagnostic challenge for clinicians. SAPHO patients were found to have high levels of the proinflammatory protein S100A12, warranting further evaluation of this protein as a biomarker [80]. S100A12, histatins, S-type cystatins, statherin, acidic proline-rich proteins, among others, were also detected in the saliva of Wilson’s disease patients, a rare inherited disorder of copper metabolism with hepatic, neurological and psychiatric symptoms that can be fatal, if not treated [81]. The salivary proteome of Wilson’s disease patients showed patterns of inflammation and oxidative stress mirroring the pathology of the disease. Specifically, certain residues in a number of proteins, such as calcium‐binding proteins, S100A9 and S100A8, were found oxidized and their identification could be used for monitoring the disease progression [81]. Celiac disease is a metabolic disease which interferes with everyday normality of individuals. Patients with celiac disease have developed intolerance in wheat proteins or gluten. A proteomic study established that there is no difference in levels of proline-rich proteins in patients with celiac disease and healthy individuals, despite their structural similarity with gluten, leaving open questions for the different mechanisms of tolerance versus immunogenicity of proline-rich proteins and gluten, respectively, in celiac disease [82].

### Neurological diseases

Saliva proteomics could be beneficial as well in the early diagnosis and monitoring of neurological conditions [83]. Autism spectrum disorders or ASD include a range of similar conditions that affect a person’s communication and behaviour and can be devastating for the person as well as its entourage. Even though data in the literature remain scarce, there are promising studies showing that saliva proteomics could complement behavioural assessments for early diagnosis and intervention, which could improve greatly the functioning of ASD sufferers. Those studies identified a decrease in proteins that regulate saliva secretion (statherin, histatin 1, and acidic proline-rich proteins) and an increase in factors known to be elevated in inflammation such as elevated prolactin-inducible protein, lactotransferrin, Ig kappa chain C region, Ig gamma-1 chain C region, Ig lambda-2 chain C regions, neutrophil elastase, polymeric immunoglobulin receptor and DMBT1 [2]. Significantly, post-translational modifications and protein misfolding seem also to play a critical role in the outcome of autism spectrum disorders and other neurological diseases or to be influenced by secondary mechanisms [84]. Oxidation, misfolding and aggregation of proteins all seem to have a causative role in the development of Parkinson’s disease as well [85]. Altogether, the analysis of proteins found in saliva in the above neurological cases pointed to alterations of proteins that act as sensors of oxidative stress.

### Perspectives

The analysis of the saliva proteome for the discovery of clinical biomarkers presents a number of challenges common in every proteomic analysis such a large dynamic range of protein concentrations, sample degradation, and variations resulting from the different quantitation methods used. Additionally, limitations in salivary biomarker validation and variations in data sets can be derived if the factors that contribute to sample composition and stability are not taken into consideration in comparative proteomic analysis. The contribution of the oral cavity microbiome to the salivary proteome is a factor that was overlooked so far and requires more attention [9]. However, saliva constitutes a less complex biological mixture than plasma and salivary proteomic analysis requires an easier, non-distressing, inexpensive sampling method with basic storage requirements of the samples. Most importantly saliva, although less stable, is a biofluid that unlike blood can be easily “spared” and therefore can play an important role in disease monitoring by replacing blood assays, even in situations where extra care is needed, such as paediatric diagnostics, if properly interpreted [43]. Thousands of samples could be collected by dentists, institutions (schools, army, health care units) and be part of nation-wide multi-centric studies like the 100,000 genomes [86] or the UK Biobank project [87]. Subsequently, national reference proteomes could contribute to large-scale validation of identified protein biomarkers and the development of clinical tests. However, for these large-scale cohorts, it is imperative to have a careful patient classification system and reliable statistical analysis. Regarding the technology used, it is important that mass spectrometry SRM/MRM approaches start delivering results with high sensitivity and specificity advancing the field of biomarker discovery in salivary proteomics. However, the combination of discovery and targeted proteomics in large cohorts that combine further different techniques, such as immune-approaches, although still scarce, deliver the most reliable and promising results [88]. In the near future, SRM platforms need to be implemented in biological and clinical labs and transformed into POC platforms [89], which together with the ‘lab-on-a-chip’ approaches will facilitate the diagnosis and monitoring of many human diseases. Many efforts are also on the way comparing serum/plasma and saliva composition and define the extent that they can mirror the same physiological or disease condition.

## Conclusions

Taken collectively, the most recent data in the literature suggest that salivary proteomics can offer many new perspectives into monitoring a substantial number of human diseases and conditions especially to the ones requiring frequent and long-term monitoring such as infectious diseases.

## Supplementary information

**Additional file 1.** Additional table.

## Data Availability

Not applicable

## Abbreviations

NIF: adjacent noncancerous fluids
SERPINA1: alpha-1-antitrypsin protein
ANXA1: annexin-1
ASD: autism spectrum disorders
cGVHD: chronic graft-versus-host disease
CFH: complement factor H
CSTB: cystatinB
DDA: data-dependent acquisition
DIA: data-independent acquisition
NIDCR: national institute dental and craniofacial research
DMBT1: deleted in malignant brain tumour 1 protein
FGA: fibrinogen alpha chain
GADPH: glyceraldehyde 3-phosphate dehydrogenase
HBV: hepatitis B virus
HCV: hepatitis C virus
HCC: hepatocellular carcinoma
HGF: hepatocyte growth factor
IL-1ra: interleukin-1 receptor antagonist
IL1RN: interleukin‐1 receptor antagonist protein
MS: mass spectrometry
MALT: mucosa-associated lymphoid tissue
MRM: multiple reaction monitoring
NID1: nidogen-1
NPY: neuropeptide Y
OSCC: oral squamous cell carcinoma
POC: point-of-care
PRM: parallel reaction monitoring
S100A2: S100 calcium‐binding protein A2
SLPI: secretory leukocyte protease inhibitor
SERPINH1: serpin H1
SS: sjogren’s syndrome
SLC3A2: solute carrier family 3 member 2
SRM: selected reaction monitoring
TPI1: triosephosphate isomerase
TIF: tumour interstitial fluids
VIP: vasoactive intestinal peptide
